# The Impact of Selective Dopamine D2, D3 and D4 Ligands on the Rat Gambling Task

**DOI:** 10.1371/journal.pone.0136267

**Published:** 2015-09-09

**Authors:** Patricia Di Ciano, Abhiram Pushparaj, Aaron Kim, Jessica Hatch, Talal Masood, Abby Ramzi, Maram A. T. M. Khaled, Isabelle Boileau, Catherine A. Winstanley, Bernard Le Foll

**Affiliations:** 1 Translational Addiction Research Laboratory, Centre for Addiction and Mental Health, University of Toronto, 33 Russell Street, Toronto, Canada M5S 2S1; 2 Alcohol Research and Treatment Clinic, Addiction Medicine Services, Ambulatory Care and Structured Treatments, Centre for Addiction and Mental Health, Toronto, ON, Canada; 3 Campbell Family Mental Health Research Institute, Centre for Addiction and Mental Health, CAMH, Toronto, ON, Canada; 4 Department of Family and Community Medicine, University of Toronto, Toronto, ON, Canada; 5 Department of Pharmacology, University of Toronto, Toronto, ON, Canada; 6 Department of Psychiatry, Division of Brain and Therapeutics, University of Toronto, Toronto, ON, Canada; 7 Institute of Medical Sciences, University of Toronto, Toronto, ON, Canada; 8 Pain Management Unit, Department of Anaesthesia, Medical Research Institute, Alexandria University, Alexandria, Egypt; 9 Addiction Imaging Research Group, Centre for Addiction and Mental Health, 250 College Street, Toronto, ON, Canada M5T 1R; 10 Department of Psychology, University of British Columbia, Vancouver, BC, Canada; University of Leicester, UNITED KINGDOM

## Abstract

Gambling is an addictive disorder with serious societal and personal costs. To-date, there are no approved pharmacological treatments for gambling disorder. Evidence suggests a role for dopamine in gambling disorder and thus may provide a therapeutic target. The present study therefore aimed to investigate the effects of selective antagonists and agonists of D2, D3 and D4 receptors in a rodent analogue of the Iowa gambling task used clinically. In this rat gambling task (rGT), animals are trained to associate different response holes with different magnitudes and probabilities of food pellet rewards and punishing time-out periods. As in the Iowa gambling task, the optimal strategy is to avoid the tempting high-risk high-reward options, and instead favor those linked to smaller per-trial rewards but also lower punishments, thereby maximizing the amount of reward earned over time. Administration of those selective ligands did not affect decision making under the rGT. Only the D4 drug had modest effects on latency measures suggesting that D4 may contribute in some ways to decision making under this task.

## Introduction

Gambling disorder is now subsumed under the ‘substance-related and addictive disorders’ section in the recently released DSM-5 [1]. Like addiction [2, 3], gambling is believed to be mediated by mesolimbic dopamine [4–6]. Indeed, pathological gambling can emerge secondary to treatment of Parkinson’s with dopamine agonists [7]. To-date, no approved pharmacological treatments exist for gambling disorder, and thus, dopamine agents may provide some efficacy in this regard.

There are 2 families of dopamine receptors, the D1-like (D1 and D5), and the D2-like (D2, D3, D4). Of these receptors, the D2 subtype has been used as a treatment for disorders such as schizophrenia, albeit with debilitating side effects [8]. Thus, the D3 and D4 subtypes may be promising as targets devoid of non-selective effects [9–11]. In this regard, both the D3 and D4 subtypes have restricted localization in the brain, consistent with a role in cognition and emotion [12, 13]. Specifically, D3 receptors are localized to the isles of Calleja, mammillary bodies, accumbens shell, frontoparietal cortex and the substantia nigra/ventral tegmental area (SN/VTA), basolateral amygdala and lateral habenula [14] [15–17], while D4 receptors are found in cerebral cortex, amygdala, hypothalamus and pituitary, sparsely in the basal ganglia [18] [19–21] [22] and the retina [23].

The role of dopamine receptors in gambling has recently been investigated in rodent models such as the rat gambling task (rGT), a paradigm that is based on the Iowa Gambling task (IGT) used clinically to investigate gambling-related decision making [24]. In this task, rats choose between different options, each associated with varying magnitudes and probabilities of gains and losses. As in the IGT, the optimal strategy is to avoid the options paired with larger per-trial rewards as these are also associated with longer punitive time-outs which limit the amount of reward earned per session. The non-selective D2/3/4 antagonist eticlopride significantly improved choice [25] on this task, increasing the choice of the option that yields the maximum pellet profits. Furthermore, administration of the D2/3/4 agonist quinpirole increased near-miss errors on a rodent slot machine task (rSMT). Interestingly, this latter deficit appears to result from quinpirole’s actions at the D4 receptor, as this drug effect could be blocked by a selective D4 antagonist and mimicked by a D4 agonist [26, 27]. However, whether the effects of D4 agents likewise impact performance of the rGT has yet to be determined. This question is of significant interest, as it may indicate the degree to which the involvement of D4 receptors is universal in multiple forms of gambling-related choice, and therefore of clinical interest for a range of gambling disorders.

The purpose of the present study was therefore to evaluate the effects of various dopamine agonists and antagonists on the rGT [24]. Due to the selectivity of the chosen ligands [28] [29] and the restricted localization of D3 and D4 receptors, the effects of the selective D3 antagonist SB 277011-A and the D4 antagonist L745, 870 were studied. In addition, the D3 and D4 agonists PD128907 and PD168077, respectively, were tested, to study whether reciprocal effects can be found as compared to the antagonists. It is hypothesized that, consistent with previous studies, D4 agonists and antagonists will have reciprocal effects on the rGT (a D4 antagonist will improve performance). The D2 antagonist L741626 was also studied to further replicate previous findings [25] that D2 antagonists improve behavior. Given the previous investigations of the effects of D2 agonists on gambling [26, 27], an exemplar of this drug class was not included here.

## Methods

### Subjects

Subjects were male Long–Evans rats (*n* = 41; Charles River Laboratories, Lachine, Quebec). All animals weighed 300–325 g at the start of the experiment. Animals were individually housed in a temperature-controlled colony room under a 12 h reverse light cycle (lights off at 7:00 A.M.). Testing took place between 09:00AM and 2:00PM, five days per week. Water was available *ad libitum* except during testing periods. Animals were food maintained on 18–20 g of standard rat chow per day, available immediately after behavioral testing. All experiments were performed in accordance with the Canadian Council of Animal Care and experimental protocols were approved by the Animal Care Committee of the Centre for Addiction and Mental Health.

### Behavioral apparatus

A detailed description of the testing chambers has been provided previously [25]. Briefly, testing took place in standard five-hole operant chambers (Med Associates, St. Albans, VT). A house light was located at the top of the chamber. Within the chambers, 5 stimulus response holes were positioned 2 cm above a metal bar floor. A stimulus light was located at the back of each hole. A food tray with a tray light at the top of the opening was located on the opposite wall. Nose-poke responses into the response holes or food tray were detected by a horizontal infrared beam. Food pellets (45 mg; Bioserv, Frenchtown, NJ) were delivered to the food tray from an external pellet dispenser. All operant chambers were contained within a ventilated and sound-attenuating box. The chambers were controlled by software written in Med-PC running on a Dell desktop computer.

### Training

Animals were trained according to previously described methods [25]. Briefly, animals were first habituated to the operant chambers and trained to make nose-poke responses into an illuminated response hole within 10 s to earn reward, similar to the training for the five-choice serial reaction time task (5CSRTT), with the exception that only 4 holes (not 5) were active, the two outer holes on each side of the array. Once animals were consistently completing 90–100 trials with ≥80% trials correct and ≤20% trial omitted, they were trained on a forced-choice version of the rGT for twelve sessions, where only one of the four possible options was presented on each trial. One option was presented in each hole and the purpose of this phase of training was for the animals to learn which response hole would be later associated with which reward and punishment outcome. Details of the rewards and punishments delivered at each hole are described below. This ensured all animals had an equal experience with the reward and punishment schedules for all options, thereby preventing the development of any simple biases toward a particular hole.

### The rGT

**Fig 1:**
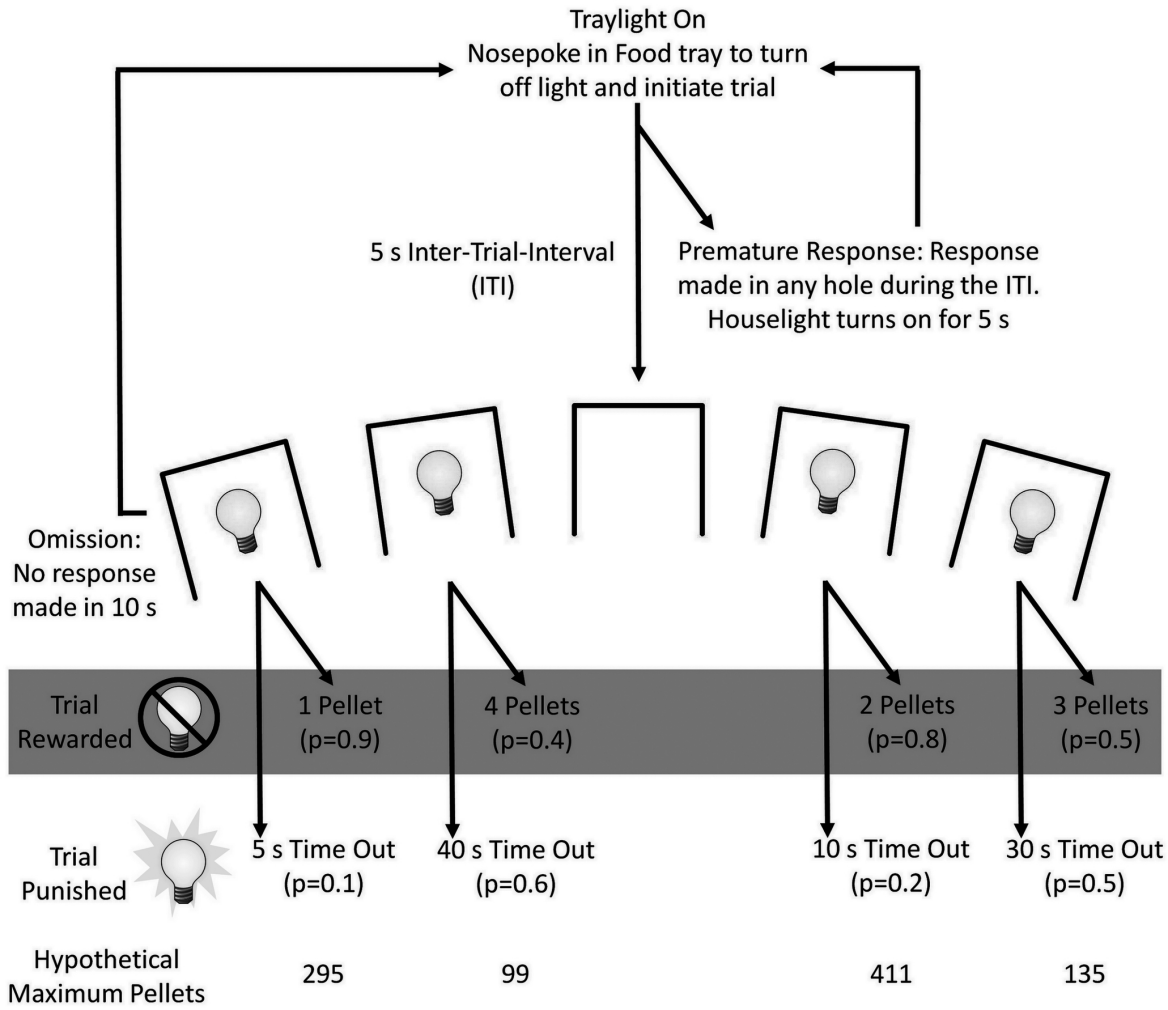
Schematic showing the trial structure of the rGT. The task began with illumination of the tray light. A nose-poke response in the food tray extinguished the tray light and initiated a new trial. After an inter-trial-interval (ITI) of 5 s, four stimulus lights were turned on in holes 1, 2, 4, and 5, and the animal was required to respond in one of these holes within 10 s. This response was then rewarded or punished depending on the reinforcement schedule for that option (indicated by the probability of a win or loss in brackets for each option). If the animal was rewarded, the stimulus lights were extinguished and the animal received the corresponding number of pellets in the now-illuminated food tray. A response at the food tray then started a new trial. If the animal was punished, the stimulus light in the corresponding hole flashed at a frequency of 0.5 Hz for the duration of the punishing timeout and all other lights were extinguished. At the end of the punishment period, the tray light was turned on and the animal could initiate a new trial. Failure to respond at the illuminated holes resulted in an omission, whereas a response during the ITI was classified as a premature response and punished by a 5-s timeout during which the house light was turned on. Taken with permission from [25].

The design of the rGT has been previously described [25] and a diagram of the trial structure and reinforcement schedules is provided in Fig 1. In brief, animals were tested once daily in a 30 min session. A trial began when a nose-poke response was made into the illuminated food tray. The tray light was subsequently turned off, initiating a 5 s intertrial interval (ITI). A response at the array during the ITI was classified as a premature response and signaled by illumination of the house light for 5 s, after which the tray light was turned on, allowing the subject to restart the trial. Following the ITI, stimulus lights within holes 1, 2, 4, and 5 were illuminated (the middle hole, hole 3, was not used for this task). If the subject did not respond at the array for 10 s, the trial was scored as an omission and the tray was subsequently illuminated, allowing the subject to start another trial. A nose-poke response in any of these holes turned off the stimulus lights and the trial would be either rewarded or non-rewarded. If the trial was rewarded, the tray light was illuminated and the appropriate number of food pellets immediately delivered into the food tray. Collection of the reward initiated the next trial. If the trial was not rewarded, no food pellets were given and the stimulus light within the chosen hole flashed at 0.5 Hz for the duration of the punishing time-out period. At the end of the punishment period, the tray light was illuminated, allowing the subject to initiate the next trial. Perseverative responses made at the array or food tray following a reward or during the time-out periods were recorded, but not punished.

Responses in the holes were rewarded such that P1 resulted in 1 pellet with a true probability of 0.9; the punishment was a 5s time out. P2 was rewarded with 2 pellets with a probability of 0.8 and the punishment was a 10s time out. P3 was rewarded with 3 pellets with a probability of 0.5 and a 30s time out signaled the punishment. For P4, this choice was rewarded with 4 pellets with a probability of 0.4 and a punishment of 40s with a 0.6 probability. These response outcomes are illustrated in Table 1. The reinforcement schedules were designed such that the two-pellet choice (P2) was optimal, in that this option would result in the most reward earned per unit time. Selection from any other option (one-, three-, or four-pellet options) yielded less reward per unit time as a consequence of the probability of winning or losing and the duration of the punishing time-out periods associated with each option. The total number of pellets possible for P1 is 295, for P2 it is 411, for P3 it is 135 and for P4 it is 99. Thus, P3 and P4 would be considered as the risky / disadvantageous choices, where the greatest reward would be associated with the highest probability and size of punishment. Selection of P1 and P2 represent a more advantageous strategy, with P2 as the best option. The location of the response holes remained stable across days

**Table 1:** Reward and punishment outcomes for the various response options in the rGT.

Group A	hole 1	hole 4	hole 5	hole 2
Group B	hole 2	hole 5	hole 4	hole 1
**Choice**	P1	P2	P3	P4
**Reward (# pellets)**	1	2	3	4
**Punishment Duration**	5s	10s	30s	40s
**Punishment Probablity**	0.1	0.2	0.5	0.6

### Drugs

Drug doses and their pharmacological action at dopamine receptors are provided in Table 2. The volume was 1 ml/kg for all drugs. All drugs were obtained from Tocris Bioscience (Ellisville, MO) with the exception of SB 277-11-A (D3 antagonist) which was obtained through Dr Steven Goldberg (NIDA-IRP, Baltimore, MD). The following drugs were dissolved in 0.9% sterile saline: L-745,870 (D4 antagonist), PD 168077 (D4 agonist) and PD 128907 (D3 agonist) while SB 277011-A (D3 antagonist) was dissolved in 10% beta-cyclodextrin in sterile water and L-741,626 in 20% Tween 80 in 0.9% sterile saline. Drugs were administered through the intraperitoneal route with the exception of L-741,626 (D2 antagonist) which was given subcutaneously. Doses and pre-treatment times were based on previous studies [25–27, 30]. All animals received all drugs and they were administered in the following consistent order: (1) L-745,870 (2) PD 168077 (3) SB 277011-A (4) L-741,626 (5) PD 128907 (Table 2). Animals were tested drug-free for a minimum of 1 week between compounds to prevent carryover effects. All drugs were prepared fresh daily, and different doses were administered according to a Latin Square design. Drug injections were given on a 3-day cycle, starting initially with a baseline session. The following day, rats received a drug or vehicle injection before testing. On the third day, animals were not tested. Injections of all agonists were given 10 min before the behavioral testing commenced, while all antagonists were given 30 min prior.

**Table 2:** Doses of agonists and antagonists used in the present study. Doses were given in counterbalanced order and the order in which the drugs were administered is given in the first column.

Order	DRUG	Effect	DOSES (mg/kg)				
2	L-741,626	D2 antagonist	Vehicle	0.1	0.3	1	N/A
1	SB 277011-A	D3 antagonist	Vehicle	0.03	1	3	N/A
5	L-745,870	D4 antagonist	Vehicle	0.5	1	5	10
3	PD 128907	D3 agonist	Vehicle	0.03	0.1	0.3	1
4	PD 168077	D4 agonist	Vehicle	0.5	1	5	10

Following initial studies, an effect of the D4 agonist PD168077 was found in the optimal rats but not the suboptimal rats (see below for explanation of optimal and suboptimal). This study was therefore repeated in a larger sample of 24 rats, 16 of which were optimal rats. These rats from the optimal subset were given 5 counterbalanced doses of the D4 agonist PD168077 (veh, 0.5, 1, 5 and 10mg/kg) i.p. 10 minutes prior to the start of the rGT session. Data were combined with the original group of rats.

### Data analysis

The percentage of trials on which an animal chose a particular option was calculated according to the following formula: number of choices of a particular option/number of total choices made × 100. To determine the percent of advantageous choices made, the percent from P1 and P2 were summed, while P3 and P4 were summed to determine the number of disadvantageous choices made.

Data were analyzed with a two-way Group (2 levels; Optimal, Suboptimal) X Dose (4 or 5 levels) mixed ANOVA with Group as the between-subjects factor, followed by *t-*tests with Bonferroni correction. Rats were divided into Optimal and Suboptimal groups based on their choice performance under vehicle; those that made more Advantageous choices under vehicle were assigned to the Optimal group. The percent of premature responses or omissions made was calculated as the number of premature responses or omissions made/total number of trials initiated × 100. The total number of trials completed was also analyzed, in addition to the latency to respond at the array and to collect reward for each choice option. Perseverative responses made during the punishment period were analyzed as a fraction of the total punishment duration experienced. Likewise, perseverative responses made after a reward was received were analyzed as a fraction of the total number of trials rewarded.

Data were analyzed with two-way Measure X Dose (4 or 5 levels) repeated-measures ANOVAs. For Choice responding, the levels of Measure were: Advantageous choice, omission and premature responses. For Latencies, the level of Measure were Choice Latency and Collect Latency, while for Perseverative Responding the levels were Punishment Perseverative Responses and Reward Perseverative Responses. The number of trials was analyzed with a one-way repeated-measures ANOVA on the effect of Dose. To determine whether the Optimal or Suboptimal subgroups were different on any measure, an addition factor of Group (2 levels) was added to the analyses. Data was analyzed separately for each drug. Significant effects were followed up with *t-*tests using the Bonferroni correction. All statistical analyses were conducted using SPSS for Windows with a criteria for significance of p<0.05.

## Results

One rat was excluded from analyses due to inactivity, so data was analyzed on 17 rats for most of the experiments. For PD168077 (D4 agonist) testing, additional experiments were conducted to determine in a larger sample if we could replicate an initial effect, so the final sample size is 33. Since Advantageous Choice and Disadvantageous choice are not independent, only the Advantageous choice is analyzed and presented. Preliminary analyses revealed that there were no group differences between the Optimal and Suboptimal groups so the data are presented as a single group. See Supporting Information for Raw values (PD168077: S1 File; L741626: S2 File; L745870: S3 File; PD128907: S4 File; SB277011A: S5 File).

### PD168077 (D4 agonist)

**Fig 2:**
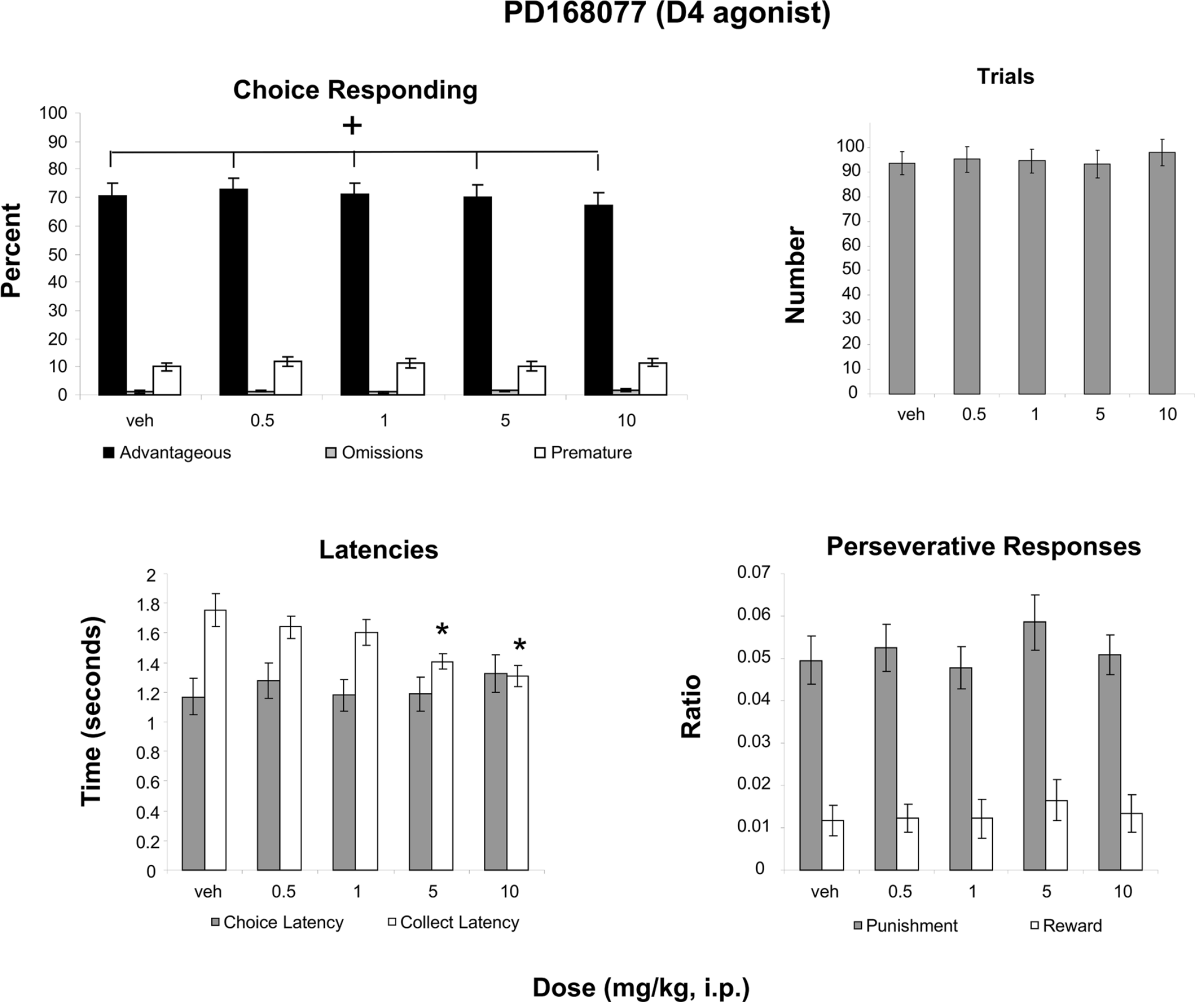
Effect of administration of a D4 agonist on the rGT. **A**: Mean ± SEM percent choice on the advantageous choice (dark bars), omissions (grey bars) and premature responses (open bars). Administration of PD168077 produced a dose-dependent decrease in advantageous responses (n = 33; F(4, 128) = 2.992, p = 0.021, one-way ANOVA). **B**: Mean ± SEM number of trials initiated. No significant effects were found. **C**: Mean ± SEM latency to make a choice (grey bars) and latency to collect the food pellets (open bars). *Differences were found between the 5mg/kg and 10 mg/kg doses when compared to vehicle (n = 33; p<0.05). **D**: Ratio ± SEM perseverative responses for punished trials (grey bars) and rewarded trials (open bars). No significant effects were found.

A two-way Measure (3 levels) X Dose (5 levels) ANOVA revealed an interaction that approached significance when corrected for non-sphericity (Fig 2; F(8, 256) = 2.155, p_GG_ = 0.073; partial eta squared = 0.063). Follow-up analyses revealed that this interaction was due to a significant effect of Dose for the Advantageous choice (F(4, 128) = 2.992, p = 0.021; partial eta squared = 0.086), but not for the other Measures (Fig 2A). *t*-Tests revealed that Advantageous choices did not differ from vehicle at any dose of PD168077. A one–way ANOVA on the effect of Dose for the number of trials (Fig 2B) revealed no effect, while a two-way Measure (Fig 2D; punishment perseveration, reward perseveration) X Dose ANOVA revealed only an effect of measure (F(1, 32) = 97.532, p<0.001; partial eta squared = .753). An ANOVA on the effect of Measure (collect latency, choice latency) X Dose (5 levels) revealed an interaction (Fig 2C; F(4, 218) = 4.944, p<0.01; partial eta squared = .134), which was due to an effect of Dose on Collect Latency (F(4, 128) = 6.811, p<0.001; partial eta squared = .175) but not Choice Latency. *t-*Tests comparing vehicle to all doses revealed a difference between vehicle and the 5mg/kg (t(32) = 3.146, p = 0.004) and 10 mg/kg doses (t(32) = 3.621, p = 0.001; Bonferroni corrected p = 0.0125).

### L745870 (D4 antagonist)

**Fig 3:**
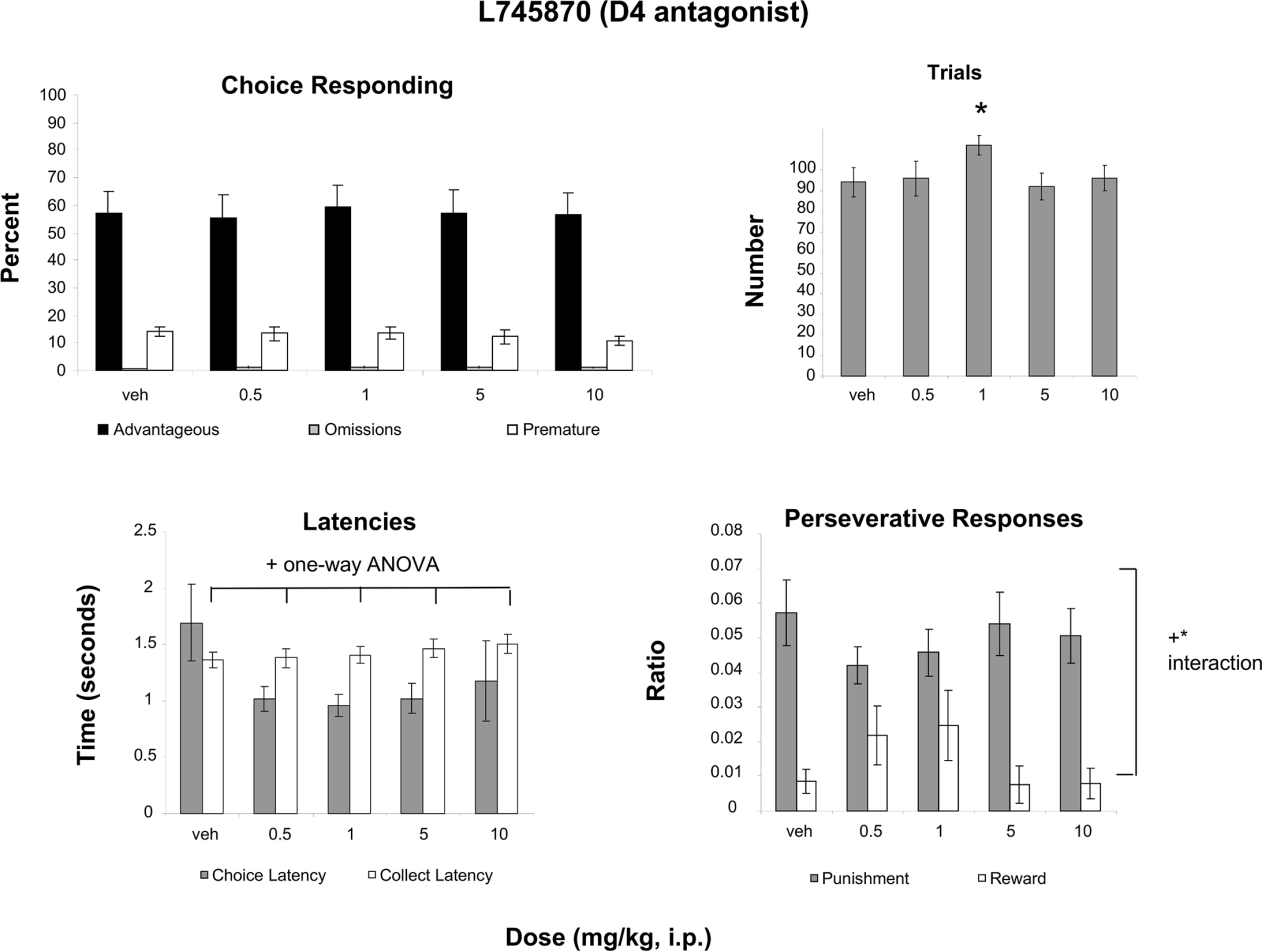
Effect of administration of a D4 antagonist on the rGT. **A**: Mean ± SEM percent choice on the advantageous choice (dark bars), omissions (grey bars) and premature responses (open bars). No significant effects were found. **B**: Mean ± SEM number of trials initiated. *Differences were found between the 1mg/kg dose and vehicle (n = 17). **C**: Mean ± SEM latency to make a choice (grey bars) and latency to collect the food pellets (open bars). Administration of L745870 produced a dose-dependent decrease in collect latency (n = 17; F(1, 15) = 3.419, p<0.05; one-way ANOVA). **D**: Ratio ± SEM perseverative responses for punished trials (grey bars) and rewarded trials (open bars). *+ A Dose X Measure interaction was revealed (F(4, 60) = 3.002, p = 0.025; n-17).

A two-way Measure (advantageous, omissions, premature) X Dose (5 levels) ANOVA revealed only an effect of Measure (Fig 3; F(2,30) = 40.972, p<0.001, partial eta squared = .732), indicating that the percentage of each measure differed but not by dose (Fig 3A). An effect of Dose was revealed for the number of trials initiated (Fig 3B; F(4, 60) = 4.248, p<0.004; partial eta squared—.221). Follow-up analyses revealed a difference between vehicle and the 1mg/kg dose, even when corrected for multiple comparisons (t(15) = -4.090, p = 0.001; corrected p of 0.0125). Analysis of latencies (Fig 3C) with a two-way repeated-measures Measure (Choice latency, Collect latency) X Dose (4 levels) ANOVA revealed a significant interaction (F(4, 60) = 3.335, p<0.05; partial eta squared = .182). Follow-up analyses revealed an effect of Dose only for collect latency (F(1,15) = 3.419, p<0.05; partial eta squared = .186); comparison of vehicle to each dose revealed no significant differences between any dose and vehicle. A two-way Measure (Fig 3D; Punishment Perseveration, Reward Perseveration) X Dose (4 levels) ANOVA revealed a significant interaction (F(4, 60) = 3.002, p = 0.025; partial eta squared = .167). Follow-up analyses revealed no significant one-way effects of Dose, indicating that the interaction was due to differences in trends of effects in the two measures.

### PD128907 (D3 agonist)

**Fig 4:**
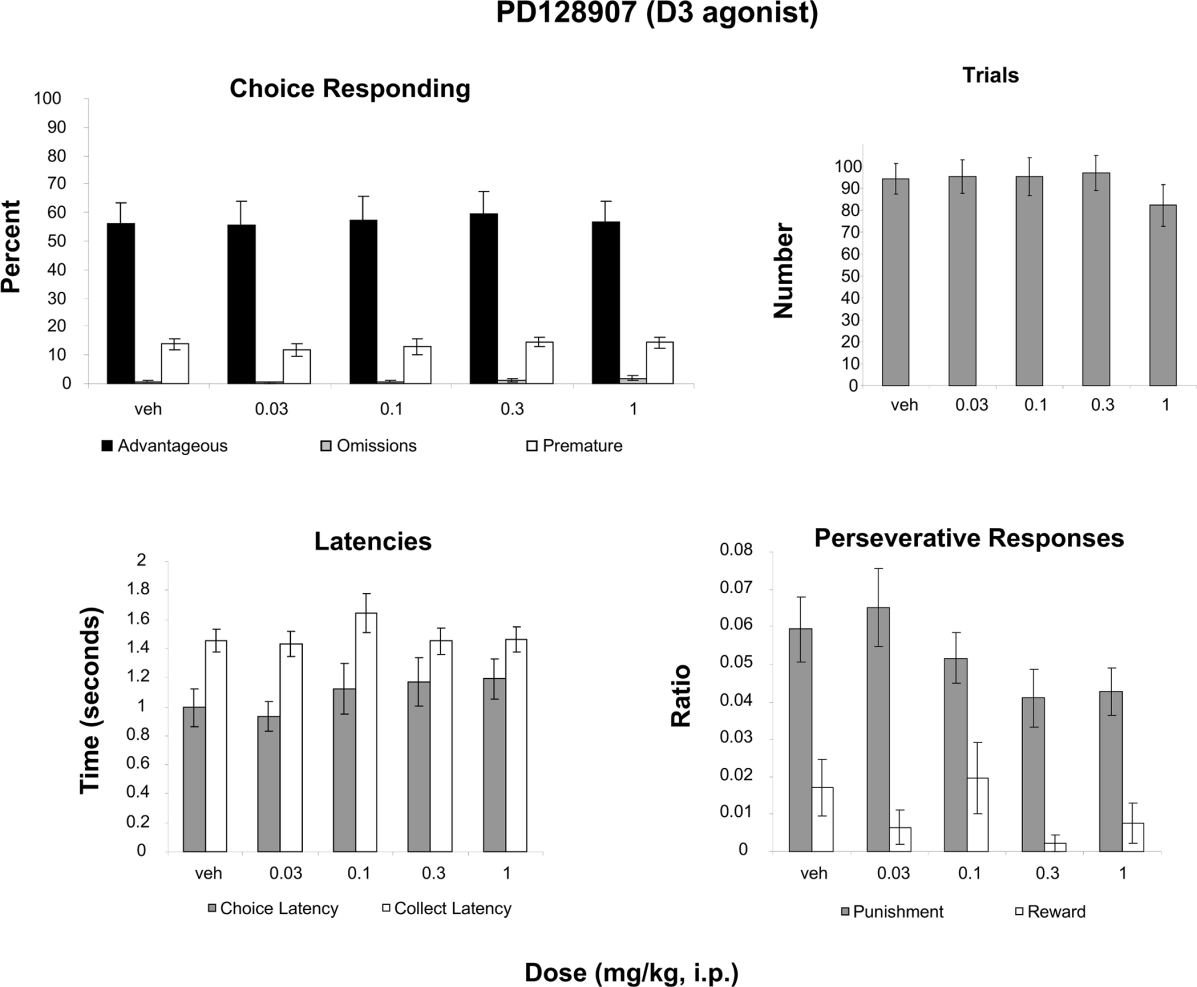
Effect of administration of a D3 agonist on the rGT. **A**: Mean ± SEM percent choice on the advantageous choice (dark bars), omissions (grey bars) and premature responses (open bars). **B**: Mean ± SEM number of trials initiated. **C**: Mean ± SEM latency to make a choice (grey bars) and latency to collect the food pellets (open bars). **D**: Ratio ± SEM perseverative responses for punished trials (grey bars) and rewarded trials (open bars). No significant differences were found for any analyses (n = 17).

A two-way Measure (advantageous, omissions, premature) X Dose (5 levels) ANOVA revealed only an effect of Measure (Fig 4; F(2,30) = 40.710, p<0.001, partial eta squared = .731), indicating that the percentage of each measure differed but not by dose (Fig 4A). No effect of Dose was revealed for the number of trials initiated (Fig 4B). Analysis of latencies (Fig 4C) with a two-way repeated-measures Measure (Choice latency, Collect latency) X Dose (4 levels) ANOVA revealed only an effect of Latency (F(1, 15) = 12.207, p<0.01; partial eta squared = .449). A two-way Measure (Punishment, Perseveration, Reward Perseveration) X Dose (4 levels) ANOVA revealed no effects (Fig 4D).

### SB277011-A (D3 antagonist)

**Fig 5:**
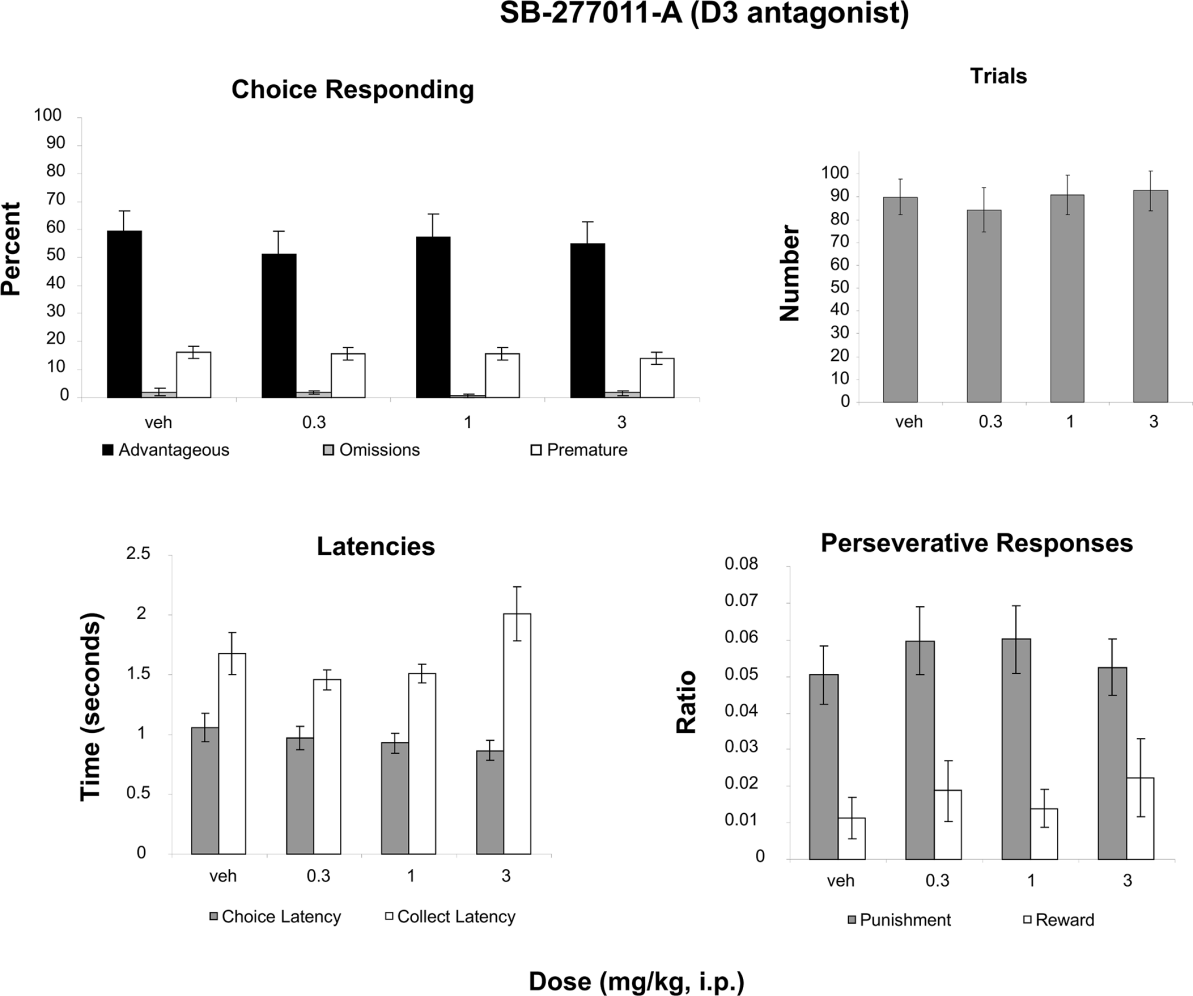
Effect of administration of a D3 antagonist on the rGT. **A**: Mean ± SEM percent choice on the advantageous choice (dark bars), omissions (grey bars) and premature responses (open bars). **B**: Mean ± SEM number of trials initiated. **C**: Mean ± SEM latency to make a choice (grey bars) and latency to collect the food pellets (open bars). **D**: Ratio ± SEM perseverative responses for punished trials (grey bars) and rewarded trials (open bars). No significant differences were found for any analyses (n = 17).

A two-way Measure (advantageous, omissions, premature) X Dose (4 levels) ANOVA revealed only an effect of Measure (Fig 5; F(2,30) = 46.418, p<0.001, partial eta squared = .756), indicating that the percentage of each measure differed but not by dose (Fig 5A). No effect of Dose was revealed for the number of trials initiated (Fig 5B). Analysis of latencies (Fig 5C) with a two-way repeated-measures Measure (Choice latency, Collect latency) X Dose (4 levels) ANOVA did not reveal any effects. A two-way Measure (Punishment Persveration, Reward Perseveration) X Dose (4 levels) ANOVA revealed only an effect of Measure (Fig 5D; F(1, 15) = 20.269, p<0.001; partial eta squared = .575).

### L741626 (D2 antagonist)

**Fig 6:**
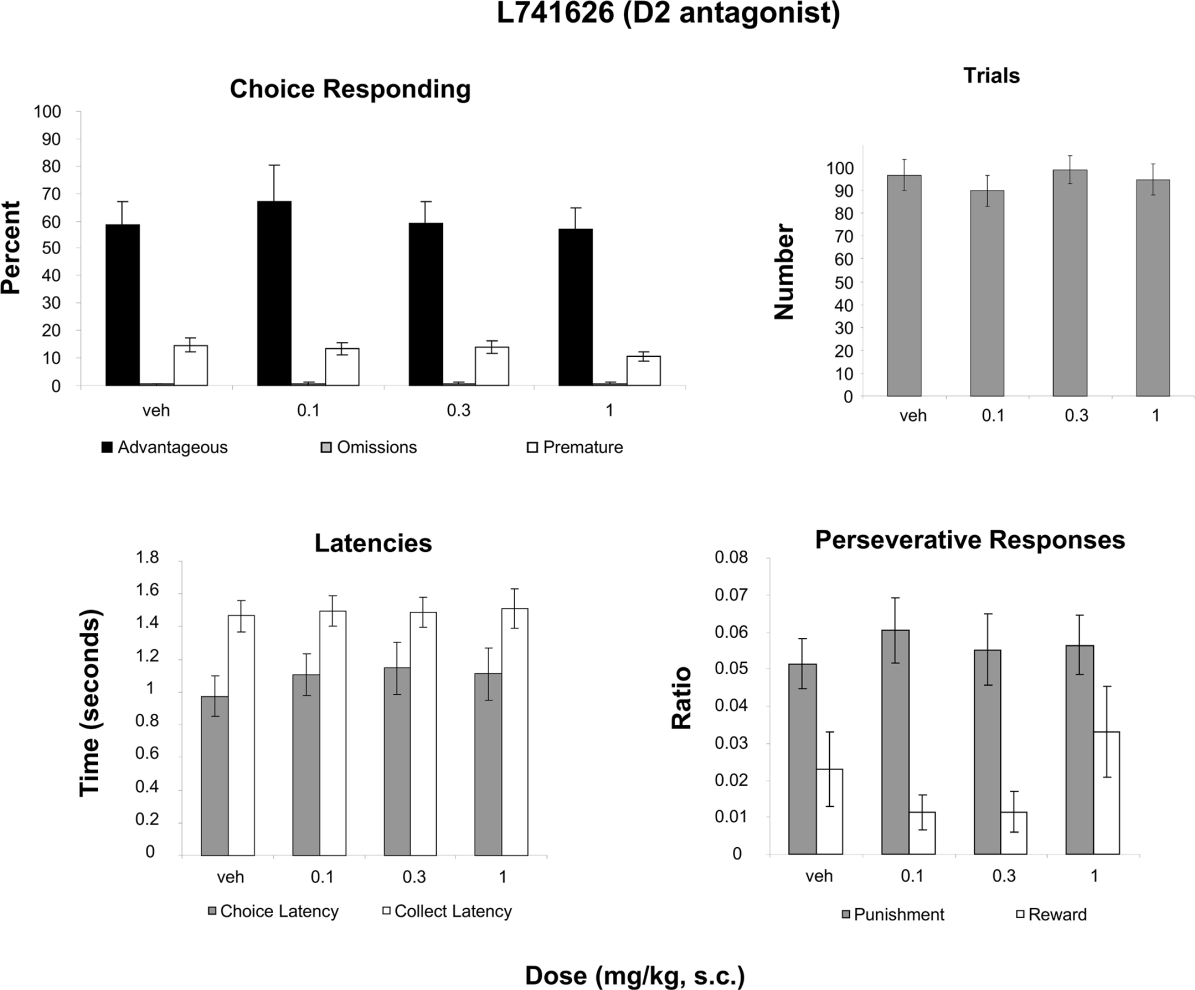
Effect of administration of a D2 antagonist on the rGT. **A**: Mean ± SEM percent choice on the advantageous choice (dark bars), omissions (grey bars) and premature responses (open bars). **B**: Mean ± SEM number of trials initiated. **C**: Mean ± SEM latency to make a choice (grey bars) and latency to collect the food pellets (open bars). **D**: Ratio ± SEM perseverative responses for punished trials (grey bars) and rewarded trials (open bars). No significant differences were found for any analyses (n = 17).

A two-way Measure (advantageous, omissions, premature) X Dose (4 levels) ANOVA revealed only an effect of Measure (Fig 6; F(2,30) = 35.158, p<0.001, partial eta squared = .701), indicating that the percentage of each measure differed but not by dose (Fig 6A). No effect of Dose was revealed for the number of trials initiated (Fig 6B). Analysis of latencies (Fig 6C) with a two-way repeated-measures Measure (Choice latency, Collect latency) X Dose (4 levels) ANOVA revealed only an effect of Latency (F(1, 15) = 7.425, p<0.05; partial eta squared = .331). A two-way Measure (Punishment, Perseveration, Reward Perseveration) X Dose (4 levels) ANOVA also revealed only an effect of Perseveration type (Fig 6D; F(1, 15) = 62.164, p<0.001; partial eta squared = .806).

## Discussion

The purpose of the present study was to evaluate the effects of agents selective for the different subtypes of D2-like receptors on the rGT. None of the agents tested markedly affected choice. The D4 agonist decreased, while the antagonist increased, latencies to collect the food pellets.

Here, the D4 agonist drug did not affect choice behavior, but there was a change in the latency to collect food pellets, which may represent an effect on motivation to receive food. However, we previously found no impact of D4 drug on food taking or food seeking [31]. Previous studies [27, 32] found that a D4 agonist increased erroneous collect responses on a slot machine task. It is unclear why the two gambling tasks are affected differently.

Consistent with findings from the slot machine task [27], D3 agonists and antagonists had no effects on the rGT. This provides converging support from animal models that D3 agents are not involved in gambling, a tenet that is somewhat surprising given the hypothesized role of D3 receptors in addictive behaviors [33–35]. It may be important to note, in this regard, that the laboratory-based tasks used in humans and rodents do not capture all aspects of pathological gambling behavior. The rGT and IGT provide a measure of how successful an individual is at “playing the odds’ and discerning the correct strategy amongst competing options, whereas slot-machine analogues detect sensitivity to near-miss phenomena. Although the rGT does provide an index of motor impulsivity in the form of premature responding, the impulsivity construct is notoriously multi-faceted, and this type of impulsivity is not sensitive to D3-selective drugs. As such, future studies will need to further investigate the exact contribution of D3 receptors to gambling.

Given previous results, it may seem challenging that there was a lack of effect of the selective D2 receptor antagonist L-741626 on the rGT. Previous reports have found that the non-selective D2 antagonist eticlopride improved performance on the rGT, as evidenced by an increase in preference for the best option [25]. However, other reports have failed to observe any effects of the ligand [36], suggesting this effect may not be as robust as it first appeared. The D2-like receptor agonists quinpirole and bromocriptine are also without effect on the rGT [25]. Interestingly, the impairment in performance caused by quinpirole on the slot machine task could not be attenuated by either eticlopride, L-741626 or the selective D3 receptor antagonist SB-277011-A, but could be ameliorated by the D4 antagonist, L-745,870 [27]. Thus, the effects of quinpirole were predominantly attributed to D4 receptors, and highlight the importance of this receptor subtype in gambling. It may be the case that the rGT is affected by drugs that target more than one type of receptor, or receptor subtype (such as eticlopride) may be effective in this task. In one report, dopamine and norepinephrine reuptake inhibition were less effective at disrupting performance on the rGT than administration of these drugs in combination [37]

It should be noted that the rGT represents choice behavior, among other behaviors, that together may model the cognitive deficits associated with gambling, aspects that are important in the diagnosis of this condition in the DSM-5. In this regard, other studies have found effects of D2 and D3 antagonists on cognitive performance that together implicate D3 receptors in cognitive abilities [38]. For example, in a novel object discrimination task, a measure of memory, it was revealed that impairments in this task were reversed by a D3 antagonist S33084, while the intact performance was impaired by the D2 antagonist L741, 626 [39], suggesting, consistent with evidence reviewed elsewhere, that D3 antagonists improve cognition [38], while D2 antagonists may impair it. Most relevant to this discussion, perhaps, was the finding that the D3 antagonist S33138 did not affect behavior on the serial reaction time task [40], a model that is most similar to the rGT used here, having similar behavioral measures with the exception that animals are not required to make choices between those that have different magnitudes of reward and probabilities of reward/punishment. Thus, it may be, that depending on the cognitive demands, D3 antagonists may not be involved in certain cognitive tasks, including those measured here. By contrast, the D2 antagonist sulpiride impaired accuracy and latency measures [41], in contrast to either the null or beneficial effects of D2 receptor antagonists reported on the rGT [25]. The exact reasons for these discrepancies need to be determined but they highlight the important and selective role of D2 and D3 receptors in different measures of cognition, which, together, may represent the combination of cognitive tasks involved in the rGT.

At first glance, it may appear that the present results are not consistent with reported findings that D4 agonists improve cognitive performance. It has been previously reported that A-412997, a selective D4 agonist, improved acquisition of an inhibitory avoidance task [42, 43], a social recognition test [42] and a novel object recognition test [44]. All these tasks, however, assess memory function, and it may be the case that the agents used in the present task do not affect memory performance as required in the rGT. Indeed, modest effects on latency to collect pellets was seen following administration of D4 agents, suggesting that any impairments that exist were not in memory, but rather, in some aspect of responding based on the outcome, food pellets. This should be considered in view of findings that heterozygous mice with a partial reduction in D4 receptors were impaired in inhibiting a response to an irrelevant stimulus in a rodent 5 choice continuous performance test [45], implicating D4 receptors in the ability to make a response option when provided with information about various stimuli.

The limitation to this study is the use of pharmacological agents that, although they are the most selective agents available at the present time, may be acting at receptor sites other than those which form the basis of interpretation of the present results. Overall our results do not support strong modulatory role for D2, D3 and D4 ligands on choice behaviors under the rGT. Future studies will be tasked with providing converging evidence in this emerging field, especially with respect to the role of the D4 receptor.

## Supporting Information

S1 File Raw values upon which conclusions are based for PD168077. (XLS)

S2 File Raw values upon which conclusions are based for L741626. (XLS)

S3 File Raw values upon which conclusions are based for L745870. (XLS)

S4 File Raw values upon which conclusions are based for PD128907. (XLS)

S5 File Raw values upon which conclusions are based for SB-277011-A. (XLS)
